# Choice of treatment for fever at household level in Malawi: examining spatial patterns

**DOI:** 10.1186/1475-2875-6-40

**Published:** 2007-04-10

**Authors:** Lawrence N Kazembe, Christopher C Appleton, Immo Kleinschmidt

**Affiliations:** 1 Applied Statistics and Epidemiology Research Unit, Mathematical Sciences Department, Chancellor College, University of Malawi, Zomba, Malawi; 2 Malaria Research Programme, Medical Research Council, Durban, South Africa; 3 School of Biological and Conservation Sciences, University of KwaZulu-Natal, Durban, South Africa; 4 Infectious Diseases Epidemiology Unit, Department of Epidemiology and Population Health, London School of Hygiene and Tropical Medicine, London, UK

## Abstract

**Background:**

Although malaria imposes an enormous burden on Malawi, it remains a controllable disease. The key strategies for control are based on early diagnosis and prompt treatment with effective antimalarials. Its success, however, depends on understanding the factors influencing health care decision making at household level, which has implications for implementing policies aimed at promoting health care practices and utilization.

**Methods:**

An analysis of patterns of treatment-seeking behaviour among care-givers of children of malarial fever in Malawi, based on the 2000 Malawi demographic and health survey, is presented. The choice of treatment provider (home, shop, or formal hospital care, others) was considered as a multi-categorical response, and a multinomial logistic regression model was used to investigate determinants of choosing any particular provider. The model incorporated random effects, at subdistrict level, to measure the influence of geographical location on the choice of any treatment provider. Inference was Bayesian and based on Markov chain Monte Carlo techniques.

**Results and Conclusion:**

Spatial variation was found in the choice of a provider and determinants of choice of any provider differed. Important risk factors included place of residence, access to media, care-giver's age and care factors including unavailability and inaccessibility of care. A greater effort is needed to improve the quality of malaria home treatment or expand health facility utilization, at all levels of administration if reducing malaria is to be realised in Malawi. Health promotion and education interventions should stress promptness of health facility visits, improved access to appropriate drugs, and accurate dosing for home-based treatments.

## Background

Although malaria imposes an enormous burden on Malawi, it remains a controllable disease. The key strategies for control are based on early diagnosis and prompt treatment with effective anti-malarials [1], which can reduce morbidity, mortality and interrupt malaria transmission [2-5]. The success of this strategy depends, however, on understanding the factors influencing health care decision making at household level, which has implications for implementing policies aimed at promoting health care practices and utilization [5-7].

In Malawi, cross-sectional studies have highlighted that most treatments for fever occur outside the formal sector [8-10], although treatment in public health facilities is free. Similar studies, carried out in Africa, reported that 60 to 80 percent of presumed cases are treated at home [11,12]. Frequently, formal health care is sought only if initial treatment fails. Health care decisions are influenced by several factors including individual, household and community factors. For instance, family members are key to successful implementation of early health care decision making [13,14]. In particular, mothers are mainly responsible for all the initial decisions and remedial actions for management of childhood diseases. Male spouse decisions are most likely associated with positive health-seeking behaviour, mainly outside the home [15]. Health care decision is also influenced by the household resource base, and availability of funds and drugs at home at the time of illness [16,17].

Community factors, for example, availability of a clinic in an area may increase the chance of visiting for people living there [18]. Self-medication in an area may be preferred because professional care may not be available, inaccessible, expensive or of poor quality [19,20]. Sociocultural factors, such as traditional beliefs, would delay seeking formal care [7]. Patterns of care will, therefore, vary from place to place. Until recently, most studies of choice of treatment considered the first two factors, i.e., individual and household characteristics [13,14]. However, there is a growing number of analyses of areal/community effects on choice of treatment differentials, mainly using multilevel models [20-22], reporting substantial areal variations in health care access and utilization, which persist after controlling for individual and household factors. Although multilevel models are able to account for areal factors, there is an important gap compared with spatial analyses. There is need to highlight areas of similar pattern, and whether there is increased or decreased risk because this will lead to identifying potential inequalities in health care access and utilisation [21,23,24]. Moreover, little consideration, so far, has been given towards understanding spatial patterns in the choice of treatment for malaria.

The purpose of this study is to quantify the spatial effect of area of residence on the choice of treatment among care-givers of children of fever in Malawi, in order to identify areas of increased or decreased risk. By highlighting the health-seeking characteristics of the population of each area, health promotion campaigns, resource allocation and improved delivery of services can be tailor-designed to the needs of the area [25]. In the present study, a small area (sub-district) analysis was carried out because any potential policy intervention is more effective when planned at local level. A unified modelling framework is presented that enables thorough investigations of the association between the choice of treatment provider, individual characteristics and areal effects. A multinomial spatial model was developed taking into account the effects of both individual and geographical factors [26].

## Methods

### Data

The data used in this analysis were collected as part of the Malawi demographic and health survey (MDHS) conducted in 2000 [27]. The 2000 MDHS is better suited to this analysis because it contains detailed geographical information that would permit spatial modelling. Moreover, the DHS is a nationally representative sample, with a relatively large number of observations on the outcome under study. The MDHS employed a two-stage sampling design. In the first stage, 560 enumeration areas (EAs) as defined in the Malawi population and housing census of 1998 were selected, stratified by urban/rural status with sampling probability proportional to the population of the EA. In the second stage, a fixed number of households were randomly selected in each EA. All women aged 15–49 were eligible for interview. A total of 13,220 women were interviewed with a response rate of 98%. An interviewer administered questionnaire was used to collect data.

In particular, data were collected, among other things, on the source of treatment, the timing of treatment, the type and dosage of treatment given for children with fever. This study analysed choice of source of treatment and investigated factors influencing the pattern among care-givers of children with fever. The alternative sources of malaria treatment were grouped into: 1) home treatment which encompassed self treatment with modern medicine, with or without prescription; 2) shops or vendors; 3) formal health facility care obtained at either public, private or mission hospital; and 4) others including traditional medicine (for example herbs) given at home, or consulting a traditional healer or no care sought. This falls naturally into a multi-categorical response variable.

**Figure 1:**
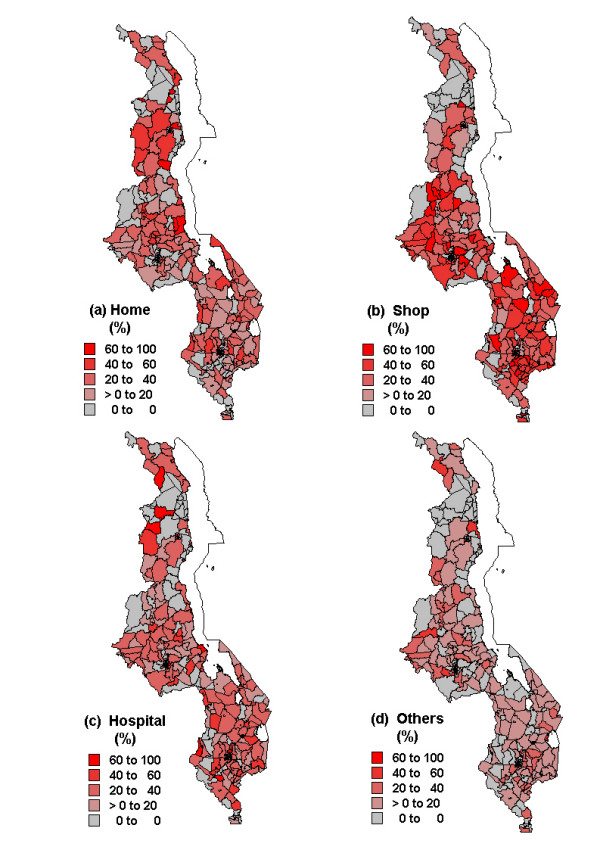
Spatial distribution of observed proportions of treatment choices made by care-givers of children with fever: (a) home treatment (b) shop treatment (c) health facility treatment (d) others (traditional/no care).

Data analysed came from 4,245 care-givers of children with fever within two weeks prior to the survey date. Because the enumeration areas were disjointed, the data were aggregated to sub-district level for spatial analysis. Although the DHS has a large and geographical dispersed sample, the number of outcomes at subdistrict were sparse (Figure 1). Smoothing techniques are required to increase precision and interpretability of spatial effects. Table 1 shows the breakdown of care-givers' choices of treatment by district. Table 2 gives the classification of care-givers' choices by individual-level covariates.

**Table 1:** Observed proportions of treatment choices among care-givers of children with fever, by districts of Malawi.

Region/District	Choice of Treatment Provider				Total
	Home (%)^†^	Shop (%)	Hospital (%)	Others^† ^(%)	*N*^‡^
*North*					
Chitipa	24.4	17.8	42.2	15.6	45
Karonga	35.1	29.2	29.5	6.3	271
Mzimba	33.3	23.1	28.2	15.4	195
Nkhatabay	40	14.7	28	17.3	75
Rumphi	50	14.3	35.7	15	26
All	34.7	24.2	30	11.2	612
					
*Central*					
Dedza	20.5	39.8	26.5	13.3	116
Dowa	36.7	32.8	20.5	10	229
Kasungu	27.5	42.9	19	10.6	273
Lilongwe	14.3	51.7	25.1	8.9	259
Mchinji	13.3	40	29.5	17.1	105
Nkhotakota	42.4	20.3	28	9.3	118
Ntcheu	26.4	34.8	30.3	8.4	178
Ntchisi	30.7	21.3	29.3	18.7	75
Salima	18.9	45.1	28	8	275
All	24.8	39.2	25.3	10.5	1678
					
*South*					
Blantyre	22.4	39	32	6.6	241
Chikwawa	15.8	34.5	36.4	13.3	165
Chiradzulu	31.8	33.3	19.7	15.2	66
Machinga	29.7	37.6	25.8	7	229
Mangochi	28.2	35.2	28.7	7.9	216
Mulanje	20.8	46.9	22.5	9.8	307
Mwanza	31.1	37.8	22.2	8.9	45
Nsanje	17.7	34.2	31.6	16.5	79
Thyolo	10.1	53.8	23.1	13	208
Zomba	18.7	41.1	28.2	12	241
Phalombe	22.7	45.5	18.2	13.6	88
Balaka	8.6	55.7	31.4	4.3	70
All	21.2	41.6	27.1	10.2	1955

Total	26.5	35.2	27.5	10.6	4245

^†^Numbers are row percentages; Others include none or traditional care;

^‡^Care-givers of children of fever.

**Table 2:** Summaries of explanatory variables included in the spatial model for the choice of treatment among care-givers of children with fever.

Variable		Choice of Treatment Provider				Total
		Home (%)	Shop (%)	Hospital (%)	Others (%)	*N*^‡^
Proportion(%)		26.9	35.0	27.5	10.6	4245

Mother/care-giver's age	<20 yr^†^	23.4	39.4	29.6	7.6	355
	20–24 yr	23.6	38.5	28.4	9.5	1301
	25–29 yr	24.4	38.4	26.6	10.6	1100
	30–34 yr	26.2	37.7	25.3	10.9	661
	35+ yr	25.4	37.5	24.5	12.6	816
Partner's education	None	23.8	40.7	21.9	13.7	644
	Primary	24.0	39.5	26.2	10.4	2795
	Secondary/Higher	27.1	32.5	32.5	7.8	719
Residence	Urban	25.5	31.6	36.6	6.3	639
	Rural	24.3	39.4	25.0	11.2	3594
Care factor: (time to facility)	Big problem	26.2	40.8	21.8	11.2	2543
	Not a problem	22.1	34.4	34.3	9.3	1690
Care factor: (availability of transport)	Big problem	26.5	40.0	21.6	11.8	2341
	Not a problem	22.0	36.0	33.2	8.7	1889
Reading newspaper	None	23.0	39.8	25.9	11.3	3136
	Once a week	25.0	36.0	29.9	9.1	800
	Daily	39.4	27.3	28.3	5.1	297
Listening to radio	None	23.8	36.3	26.7	13.2	1040
	Once a week	22.1	41.4	25.2	11.4	1037
	Daily	26.0	37.7	27.6	8.7	2156
Watch TV	None	24.7	38.4	26.2	10.8	3910
	Once a week	21.7	38.9	33.2	6.1	244
	Daily	25.3	29.1	38.0	7.6	79
Visited hospital (last 12 months)	No	29.1	36.0	21.7	13.2	1109
	Yes	22.9	39.0	28.6	9.5	3124
Toilet type	Flush	29.6	23.5	40.7	6.2	81
	Pit	24.1	39.0	27.1	9.9	3278
	None	25.1	36.8	24.6	13.4	276
Ethnicity	Chewa	25.0	40.2	23.8	11.0	1322
	Tumbuka	30.4	28.6	27.4	13.6	339
	Lomwe	19.8	44.3	25.6	10.3	749
	Tonga	39.8	18.2	29.5	12.5	530
	Yao	25.6	38.57	26.9	8.9	208
	Sena	14.9	40.9	32.2	12.0	451
	Ngoni	30.6	32.0	32.7	4.8	259
Household size	≤ 5	22.3	40.1	27.7	9.9	768
	6–10	24.3	37.9	27.3	10.5	2603
	11+ members	27.3	37.5	24.4	10.9	862

^†^Numbers are row percentages; ^‡^Care-givers of children with fever.

### Statistical analysis

#### Model

The choice of treatment provider was modelled using the multinomial logit model within the framework of discrete choice models [26,28]. The following four-categorical response variable, *Y*_*ij*_, for sources of treatment, was defined as:

Yij={1home care with modern medicine2shops or vendors3health facility4others (traditional/no care),
 MathType@MTEF@5@5@+=feaafiart1ev1aaatCvAUfKttLearuWrP9MDH5MBPbIqV92AaeXatLxBI9gBaebbnrfifHhDYfgasaacH8akY=wiFfYdH8Gipec8Eeeu0xXdbba9frFj0=OqFfea0dXdd9vqai=hGuQ8kuc9pgc9s8qqaq=dirpe0xb9q8qiLsFr0=vr0=vr0dc8meaabaqaciaacaGaaeqabaqabeGadaaakeaacqWGzbqwdaWgaaWcbaGaemyAaKMaemOAaOgabeaakiabg2da9maaceqabaqbaeaabqGaaaaabaGaeGymaedabaGaeeiAaGMaee4Ba8MaeeyBa0MaeeyzauMaeeiiaaIaee4yamMaeeyyaeMaeeOCaiNaeeyzauMaeeiiaaIaee4DaCNaeeyAaKMaeeiDaqNaeeiAaGMaeeiiaaIaeeyBa0Maee4Ba8MaeeizaqMaeeyzauMaeeOCaiNaeeOBa4MaeeiiaaIaeeyBa0MaeeyzauMaeeizaqMaeeyAaKMaee4yamMaeeyAaKMaeeOBa4MaeeyzaugabaGaeGOmaidabaGaee4CamNaeeiAaGMaee4Ba8MaeeiCaaNaee4CamNaeeiiaaIaee4Ba8MaeeOCaiNaeeiiaaIaeeODayNaeeyzauMaeeOBa4MaeeizaqMaee4Ba8MaeeOCaiNaee4CamhabaGaeG4mamdabaGaeeiAaGMaeeyzauMaeeyyaeMaeeiBaWMaeeiDaqNaeeiAaGMaeeiiaaIaeeOzayMaeeyyaeMaee4yamMaeeyAaKMaeeiBaWMaeeyAaKMaeeiDaqNaeeyEaKhabaGaeGinaqdabaGaee4Ba8MaeeiDaqNaeeiAaGMaeeyzauMaeeOCaiNaee4CamNaeeiiaaIaeiikaGIaeeiDaqNaeeOCaiNaeeyyaeMaeeizaqMaeeyAaKMaeeiDaqNaeeyAaKMaee4Ba8MaeeOBa4MaeeyyaeMaeeiBaWMaee4la8IaeeOBa4Maee4Ba8MaeeiiaaIaee4yamMaeeyyaeMaeeOCaiNaeeyzauMaeiykaKIaeiilaWcaaaGaay5Eaaaaaa@AA72@

for care giver *j *in area *i*. The response *Y*_*ij *_is considered as a realization of some latent variable *U*_*ij *_= *η*_*ij *_+ *ε*_*ij*_, which the care giver *j *maximizes, where *η*_*ij *_is the predictor and *ε*_*ij *_is the error term. A care giver chooses treatment provider category *r*, if that choice offers maximum benefits based on the principle of random maximum utility [26,28]. These benefits could be in terms of time constraints, transport cost, perceived quality of care in case of visiting a health facility or other associated opportunity costs.

The choice of provider *r*, is modelled as the probability of selecting that provider category against some reference category, with *Y*_*ij *_assumed to arise from a multinomial distribution. The influence of covariates are modelled using a multinomial logistic regression,

ηij(r)=β(r)vij+si(r),r=1,2,3
 MathType@MTEF@5@5@+=feaafiart1ev1aaatCvAUfKttLearuWrP9MDH5MBPbIqV92AaeXatLxBI9gBaebbnrfifHhDYfgasaacH8akY=wiFfYdH8Gipec8Eeeu0xXdbba9frFj0=OqFfea0dXdd9vqai=hGuQ8kuc9pgc9s8qqaq=dirpe0xb9q8qiLsFr0=vr0=vr0dc8meaabaqaciaacaGaaeqabaqabeGadaaakeaaiiGacqWF3oaAdaqhaaWcbaGaemyAaKMaemOAaOgabaGaeiikaGIaemOCaiNaeiykaKcaaOGaeyypa0Jae8NSdi2aaWbaaSqabeaacqGGOaakcqWGYbGCcqGGPaqkaaGccqWG2bGDdaWgaaWcbaGaemyAaKMaemOAaOgabeaakiabgUcaRiabdohaZnaaDaaaleaacqWGPbqAaeaacqGGOaakcqWGYbGCcqGGPaqkaaGccqGGSaalcqWGYbGCcqGH9aqpcqaIXaqmcqGGSaalcqaIYaGmcqGGSaalcqaIZaWmaaa@4DB3@

with the last choice assigned as a reference category, in order to compare choice of any provider of modern biomedical care against others including traditional or no care. Covariates are given by *v*_*ij*_, and *β*^(*r*) ^is the corresponding vector of regression parameters for choice category *r*, such that exp(*β*^*r*^) is the relative risk ratio (RRR), and si(r)
 MathType@MTEF@5@5@+=feaafiart1ev1aaatCvAUfKttLearuWrP9MDH5MBPbIqV92AaeXatLxBI9gBaebbnrfifHhDYfgasaacH8akY=wiFfYdH8Gipec8Eeeu0xXdbba9frFj0=OqFfea0dXdd9vqai=hGuQ8kuc9pgc9s8qqaq=dirpe0xb9q8qiLsFr0=vr0=vr0dc8meaabaqaciaacaGaaeqabaqabeGadaaakeaacqWGZbWCdaqhaaWcbaGaemyAaKgabaGaeiikaGIaemOCaiNaeiykaKcaaaaa@32C2@ are subdistrict-specific spatial effects for choice *r*. The random effects can be split into two components, i.e., spatially structured variation and unstructured heterogeneity. This reflects the fact that unobserved risk factors may be area-specific or may be shared or similar across neighbouring areas.

#### Analysis

Due to numerous risk factors recognized in the literature, single-variable models were fitted to identify candidate variables to include in the spatial model. These models were fitted using the maximum likelihood approach in R statistical system [29]. All variables significant at *p *= 0.20 were included in the spatial multinomial logistic model. Spatial analysis followed a Bayesian approach, with prior distributions specified for all parameters in equation (1). For the fixed parameters, *β*, diffuse priors were assumed. For the spatially structured effects, we chose the conditionally autoregressive (CAR) priors [30]. The CAR priors define areas as neighbours if they share a common boundary and neighbouring areas are assumed to have similar patterns, such that the mean of area *i *is assumed to be an average of neighbouring areas, with variance as a function of number of neighbours and spatial variance. Further, the spatial variance was assigned an inverse Gamma prior. The unstructured heterogeneity term was assumed to follow an exchangeable normal prior. Model estimates were derived by drawing samples from the posterior distribution using Markov Chain Monte Carlo (MCMC) techniques. Model implementation was carried out in BayesX 1.14 [31]. A total of 35,000 iterations were carried out, with 5,000 burn-in and thinned every 10th observation for parameter estimation. Convergence was assessed through autocorrelation functions and trace plots.

## Results

### Descriptive summaries

Of the 4,245 (30% of total sample) care-givers of children with fever, 35% obtained drugs over the counter, followed by visiting a health facility (28%), and home medicine (27%). Traditional medicines were preferred by 4.3%, while another 6.4% did not seek care. Table 1 indicates geographical variation, at district level, in the choices of treatments among care-givers. Regional variation was also notable, with the southern region having the highest proportion seeking care from the shops, while the northern region showed the highest proportion chose hospital and home care. The proportions of care-givers who sought traditional/no care were equally similar between regions, although within region variations were evident in the central and southern region. The number of observations per sub-district were relatively sparse. Observed proportions in each provider category are given in Figure 1. The mean number of observations per area was 5 (range: 0–30), 8 (range: 0–48), 5 (range: 0–28) and 3 (0–10) for home, shop, hospital and traditional/no care alternatives respectively. It is also evident that choice of treatment varied with individual characteristics (Table 2). For instance, choices differed with socio-economic factors such as access to media for all categories, demographical factors such as care-givers' age, although this was not very apparent for home-remedy category.

### Socio-economic and behavioural determinants

Table 3 gives the RRR estimated from the spatial multinomial logit model. The results show that, the relative risk of home treatment, shop and hospital care versus traditional or no care were 1.37, 1.44, 1.42 respectively for mothers of age less than 20 years compared to mothers aged 35 years. No differences in relative risk was observed between each of the three sources of treatment versus traditional or no care, for mothers with ages between 20 to 35 compared to mothers above 35 years.

**Table 3:** Relative risk ratio (95% confidence intervals) for the spatial multinomial logistic regression fitted for the choice of treatments at household level

Variable		Choice of Treatment Provider		
		Home vs others	Shop vs others	Hospital vs others^‡^
Mother/care-giver's age	<20 yr	1.37 (1.05,1.80)*	1.44 (1.12,1.86)*	1.42 (1.09,1.84)*
	20–24 yr	0.98 (0.84,1.15)	0.99 (0.85,1.15)	0.98 (0.84,1.14)
	25–29 yr	0.95 (0.81,1.11)	0.89 (0.77,1.03)	0.94 (0.81,1.10)
	30–34 yr	0.95 (0.79,1.14)	0.91 (0.77,1.09)	0.91 (0.75,1.09)
	35+ yr	1.00	1.00	1.00
Partner's education	None	0.84 (0.72,0.98)*	0.88 (0.77,1.01)	0.75 (0.65,0.88)*
	Primary	1.13 (0.96,1.33)	1.04 (0.89,1.22)	1.25 (1.06,1.47)*
	Secondary/Higher	1.00	1.00	1.00
Residence	Urban	1.20 (1.05,1.36)*	1.13 (0.99,1.28)*	1.31 (1.16,1.49)*
	Rural	1.00	1.00	1.00
Care factor: -time to facility	Big problem	1.06 (0.96,1.17)	1.07 (0.97,1.17)	0.87 (0.79,0.96)*
	Not a problem	1.00	1.00	1.00
Care factor: -transport availability	Big problem	0.99 (0.89,1.09)	0.89 (0.81,0.98)*	0.81 (0.73,0.89)*
	Not a problem	1.00	1.00	1.00
Reading newspaper	None	1.00	1.00	1.00
	Once a week	0.79 (0.67,0.94)*	0.88 (0.74,1.04)	0.91 (0.77,1.09)
	Daily	1.92 (1.49,2.48)*	1.26 (0.97,1.63)*	1.28 (0.98,1.66)*
Listening to radio	None	1.00	1.00	1.00
	Once a week	0.90 (0.81,1.01)*	1.02 (0.92,1.14)	0.93 (0.83,1.04)*
	Daily	1.28 (1.16,1.42)*	1.17 (1.06,1.29)*	1.15 (1.04,1.27)*
Watch TV	None	1.00	1.00	1.00
	Once a week	1.27 (0.91,1.77)	1.40 (1.02,1.93)*	1.50 (1.09,2.06)*
	Daily	0.72 (0.45,1.13)	0.87 (0.56,1.34)	0.77 (0.50,1.19)
Visited hospital (last 12 months)	No	1.00	1.00	1.00
	Yes	0.72 (0.45,1.13)	1.17 (1.08,1.26)*	1.26 (1.16,1.37)*
Ethnicity	Chewa	0.95 (0.81,1.11)	1.11 (0.95,1.28)	0.82 (0.70,0.95)*
	Tumbuka	0.75 (0.59,0.95)*	0.60 (0.48,0.77)*	0.63 (0.49,0.80)*
	Lomwe	0.74 (0.61,0.90)*	1.22 (1.02,1.46)*	0.90 (0.75,1.09)
	Tonga	1.10 (0.74,1.65)	0.41 (0.26,0.63)*	0.74 (0.49,1.13)
	Yao	1.18 (0.94,1.47)	1.27 (1.02,1.57)*	1.13 (0.91,1.42)
	Sena	0.54 (0.39,0.74)*	1.09 (0.82,1.45)	1.11 (0.83,1.50)
	Ngoni	1.00	1.00	1.00
Household size	≤ 5	1.12 (0.84,1.49)	1.16 (0.89,1.52)	1.73 (1.29,2.32)*
	6–10	1.09 (0.82,1.45)	1.05 (0.80,1.37)	1.74 (1.30,2.33)*
	11+	1.00	1.00	1.00

*significant at 5%; ^‡ ^others include none or traditional care

The likelihood of seeking home care compared to no or traditional care was lower for care givers whose partners had no formal education relative to those with secondary education or higher. Similarly, the probability of choosing hospital care compared to no or traditional care was lower for those with partners of no formal education relative to those with partners with secondary or higher education. However, those with partners who had at least primary education relative to those with at least secondary or higher were more likely to choose hospital care compared to no or traditional care.

Urban care givers were found more likely to choose home treatment, shop or hospital treatment compared to no or traditional care, relative to rural residents. Those who had difficulties with time needed to go to the health facility were indeed less likely to choose hospital care compared to no or traditional care. No differences were observed between home care or shop provider versus no/traditional care for those who were unwilling to take the time to go to the facility relative to those who had no problem. Similar patterns were observed for care givers who questioned the availability of transport relative to those who did not, such that those finding difficulties with transport were less likely to choose hospital care or get medicine from shops compared to no or traditional care.

Access or exposure to the media was also important in explaining the choice of health provider. Those who read newspapers at least once a week relative to not at all were less inclined to choose home care compared to no or traditional care. But those who managed to read newspapers daily were 92% more likely to choose home care compared to no/traditional care. Similarly, the relative risk of shop or hospital versus no/traditional care were 1.26 and 1.28 respectively, for those reading newspapers daily compared not at all. Listening to the radio daily increased the chance of choosing modern care (either from home, shops or hospitals) compared to no or traditional care. Similarly, those who watched television at least once a week relative to those who never watched were more likely to choose modern care from shops or hospital compared to no or traditional care. However, those listening to the radio once a week were somewhat less likely to choose home treatment or shop treatment versus traditional/no care, relative to those who did not listen at all.

The results also indicate that care-givers who usually visit a health facility at least once a year, were more inclined to choose hospital care or shop treatment compared to traditional or no care, relative to those who did not. Ethnic differences were also associated with the type of care chosen. In some instances, the likelihood of choosing any provider versus no or traditional provider was lower, and in others it was higher. For example, relative to the Ngonis, the Tumbukas, Senas and Lomwes were less likely to choose home treatment, while the Tumbukas and Tongas were less likely to get treatment from shops, and the Chewas and Tumbukas were less inclined to visit a hospital for treatment. On the other hand, compared to the Ngonis, the Yaos were more inclined towards having drugs from shops than traditional medicine or no care at all. Household size also had an effect on the choice of treatment provider. Households of size five or less and those of 6 to 10 members, relative to 11 or more members, were likely to choose hospital care compared to traditional or no care.

### Spatial effects on choice of malaria treatment

**Figure 2:**
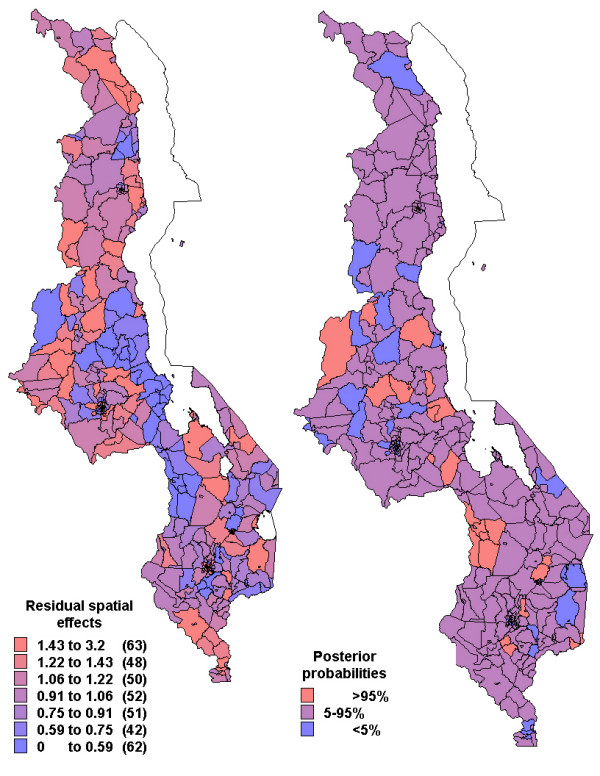
Residual spatial effects at sub-district level (1. home treatment versus no/traditional treatment). Shown are the relative risk ratio (RRR) on the left map. Right map shows corresponding posterior probabilities of RRR> 1: < 5 per cent blue, 5–95 per cent pink, >95 per cent red.

**Figure 3:**
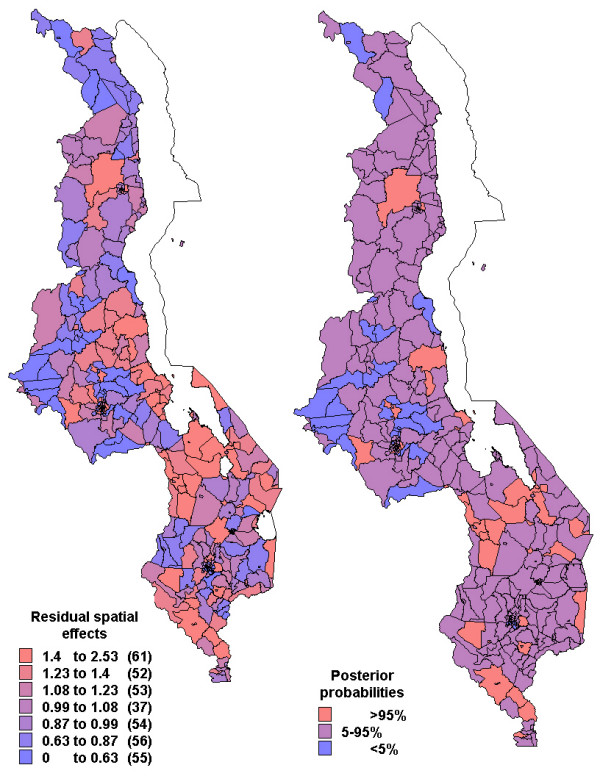
Residual spatial effects at sub-district level (2. shop treatment versus no/traditional treatment). Shown are the relative risk ratio (RRR) on the left map. Right map shows corresponding posterior probabilities of RRR> 1: < 5 per cent blue, 5–95 per cent pink, >95 per cent red.

**Figure 4:**
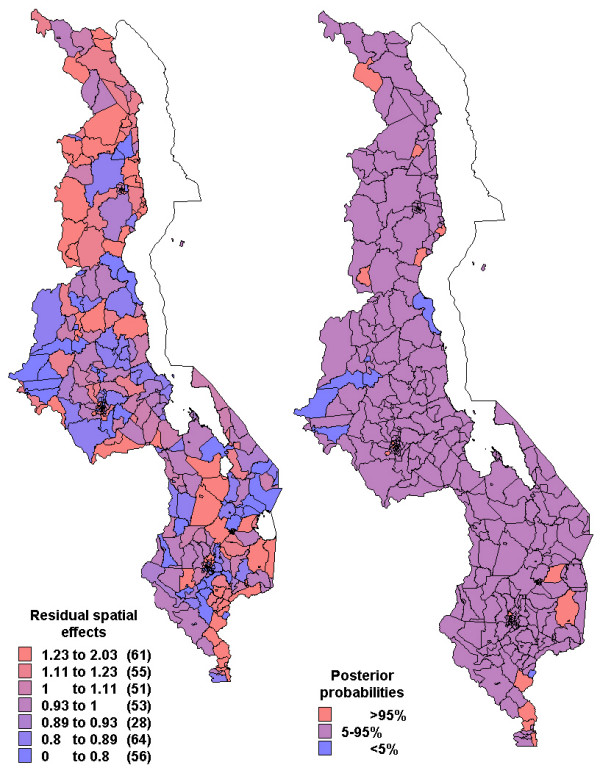
Residual spatial effects at sub-district level (3. health facility treatment versus no/traditional treatment). Shown are the relative risk ratio (RRR) on the left map. Right map shows corresponding posterior probabilities of RRR> 1: < 5 per cent blue, 5–95 per cent pink, >95 per cent red.

Figures 2 to 4 show the residual spatial variation in choice of health provider at sub-district level in Malawi, after adjusting for all factors given in Table 3. The red (blue) colour shows an increased (decreased) RRR for a particular choice versus no/traditional treatment provider. There was evidence of spatial variation in the choice of home and shops as source of treatment, but little variation for the choice of health facility care. The accompanying maps show the posterior probabilities for assessing the significance of the RRR estimate per area (sub-district), i.e, for identifying areas of excess variation compared to the overall mean (RRR = 1).

Specifically, the likelihood of getting home treatment, relative to traditional or no care, increased in central region and parts of northern region (red colour in Figure 2) while decreased along the lakeshore and parts of the southern region (blue colour). Again care-givers in the central region and parts of northern region were less likely to get antimalarial drugs from the shop, while those in the southern region were more likely to use shops as a source of antimalarial treatment (Figure 3). The probability of choosing hospital treatment versus traditional/no care was slightly higher in the northern region compared to the other areas (Figure 4). However, there is little evidence of any spatial variation, as indicated by the posterior probabilities.

## Discussion

This study was concerned with understanding the determinants of health care decisions at household level in Malawi. Although, there is a considerable literature on care-seeking behaviour in Malawi [8,10,32], this contributed to the literature in one way. The study examined geographical variations in the choices of treatment provider made by care-givers, viz: (i) home (ii) shopkeepers (iii) health facilities (iv) others: traditional healers, village health workers – in a way highlighting areas that may need further attention. This was achieved by fitting a multinomial regression model that incorporated both individual characteristics and spatially distributed random effects in a unified framework to assess excess risk at sub-district level for each health provider chosen.

The results revealed spatial variation in the choices of source of treatment, as indicated by Figures 2 to 4, having adjusted for socio-economical and behavioural factors. This pattern was very substantial for home and shop-rendered care versus traditional/no care, but slightly small for health facility care versus traditional/no care.

Factors contributing to this pattern are a matter of conjecture. Unmeasured socio-economic differences might be some of the factors related to this pattern. Studies have found that low income groups are likely to engage in self-diagnosis [12,33]. However, high socio-economic groups may also engage in self-treatment more often as reported in other studies [34], leading to high probability of self-medication in urban areas, for example the high RRR observed in the capital city might be influenced by this factor.

Other studies have found that sociocultural factors are associated with health beliefs for malaria [2,35]. For example, belief that certain fever is treatable at home might possibly influence use of home care. In other instances, communities offer supportive treatment and use home drug stocks. Variations in such cultural practices may exhibit spatial similarities within some areas and differences between others through-out the country.

The nearly similar spatial patterns of seeking formal health facility care versus no/traditional care (Figure 4), simply means that residual variation was not spatial. This suggest that most of the variation in the outcome was explained by the individual-level characteristics, some of which are factors associated with inaccessibility of formal health care across the country. Access can be impeded by time constraints, lack and cost of transportation, money for care, competing priorities at home such as child care, food preparation and formal work [10]. Health facility characteristics are also said to influence the decision to seek formal health facility care [19,36]. Quality of care (e.g. unavailability or stock-out of effective treatment, long queues) is more likely to discourage households to seek care at health facilities, which may also lead to bypassing certain health posts [20]. Differences in access to health care or quality of care may thus effect different patterns in health care utilization, inducing spatial clustering in health care utilization [19].

This analysis found that self-treatment with drugs obtained from homes or shopkeepers/vendors was very high. About 54% took medicines with or without prescription, because it is convenient to buy in shops nearby or from their home rather than going to health posts. Studies have documented that 50% of antimalarial drug use occur outside the formal health facilities [37]. A nationwide survey carried out in Malawi in 1992 found that similar proportion received medicine at home, either obtained from nearby shops or obtained earlier from health facilities [32]. In fact, home based or shop/vendor care may be more prompt than having care from elsewhere [38]. This might explain the increased likelihood of home and shop care versus traditional or no care, observed in the central region and isolated parts of the southern region (Figures 2 and 3).

The results also revealed that, as in most least developing countries, the level of health care utilization is relatively low. Only 28% of children who had fever were taken for formal curative care. This agrees with previous studies conducted in Malawi. Wirima [8] and Ettling *et al*. [39] found that prompt treatment at formal health care was accessible for only a small number of children. Similar findings have been found in a number of developing countries. For example, a study in western Thailand found that only 20% of the population had access to medical care and high proportions of self-medication [40]. In Africa it was found that drug vendors offered services closer to home and at a cheaper cost [14,41,42], thus creating convenience for care givers in terms of time and cost of travel to health facilities. Holtz *et al *[10] reported a different finding, and indicated that 58% of children in Blantyre, Malawi visited a health facility at some point during their illness.

The choice of treatment and the decision to seek care depended on a number of socioeconomic, demographic and behavioural factors including mothers age, partner's education, place of residence, assets such as radio, and access to media. These findings are similar to earlier studies [12,17]. Factors such as education and assets were the measure of income used here because DHS surveys do not collect direct data on income. Their inclusion in the model, therefore, controlled for income differentials [20]. The findings that urban households were more likely than rural ones to use formal curative care are consistent with results elsewhere [17,36,43]. Similarly, access to the media by frequently reading newspapers, listening to radio and watching television influenced the propensity to use curative care compared to no or traditional care. Although the DHS did not capture the degree of illness, this is also an important indicator of health care decisions. Mere high temperatures are likely to be treated at home first [12]. In contrast, severe malaria is often rushed to hospitals [44], but this might take place only after the first attempt at cure has failed.

To conclude, it should be emphasized that a greater effort is needed to improve the quality of malaria home treatment or expand health facility utilization, at all levels of administration if the goal of reducing malaria burden is to be realised in Malawi. Health promotion and education interventions should stress promptness of health facility visits, improved access to appropriate drugs and accurate dosing for home-based treatments [4].

## Authors' contributions

LNK conceptualized, analyzed and drafted the manuscript. IK and CCA participated in the conception, and critical review of the manuscript. All authors read and approved the final manuscript.

## References

[B1] WHO (2004). Roll back malaria technical strategies.

[B2] Hausmann-Muela S (2000). Community understanding of malaria, and treatment seeking behaviour, in a holoendemic area of southeastern Tanzania. PhD Thesis.

[B3] WHO/TDR (2003). The behavioural and social aspects of malaria and its control.

[B4] WHO (2004). Scaling up home-based management of malaria: from research to implementation.

[B5] Pagnoni F, Convelbo N, Tiendrebeogo J, Cousens S, Esposito F (1997). A community-based programme to provide prompt and adequate treatment of presumptive malaria in children. Trans R Soc Trop Med Hyg.

[B6] Fosu G (1994). Childhood morbidity and health services utilization: Cross-national comparisons of user-related factors from DHS data. Soc Sci Med.

[B7] MacCormack CP (1984). Human ecology and behaviour in malaria control in tropical Africa. Bull World Health Organ.

[B8] Wirima JJ (1996). A nation-wide malaria knowledge, attitudes and practices survey in Malawi. Trop Med Parasitol.

[B9] Government of Malawi (2002). Malaria policy.

[B10] Holtz TH, Kachur SP, Marum LH, Mkandala C, Chizani N, Roberts JM, Macheso A, Parise ME (2003). Care seeking behaviour and treatment of febrile illness in children aged less than five years: a household survey in Blantyre District, Malawi. Trans R Soc Trop Med Hyg.

[B11] Ahorlu CK, Dunyo SK, Afari EA, Koram KA, Nkrumah FK (1997). Malaria-related beliefs and behaviour in southern Ghana: implications for treatment, prevention and control. Trop Med Int Health.

[B12] Kofoed PE, Rodrigues A, Co F, Hedegaard K, Rombo L, Aaby P (2004). Which children come to the health centre for treatment of malaria?. Acta Trop.

[B13] McCombie SC (1996). Treatment seeking for malaria: a review of recent research. Soc Sci Med.

[B14] McCombie SC (2002). Self-treatment for malaria: the evidence and methodological issues. Health Policy Plan.

[B15] Mwenesi H, Harpham T, Snow RW (1995). Child malaria treatment seeking practices among mothers in Kenya. Soc Sci Med.

[B16] Miguela CA, Tallo VL, Manderson L, Lansang MA (1999). Local knowledge and treatment of malaria in Agusan del Sur, The Philippines. Soc Sci Med.

[B17] Uzochukwu ESC, Onwujekwe OE (2004). Socio-economic differences and health seeking behaviour for the diagnosis and treatment of malaria: a case study of four local government areas operating the Bamako initiative programme in south-east Nigeria. Int J Equity Health.

[B18] Stephenson R, Baschieri A, Clements S, Hennink M, Madise N (2006). Contextual influences on the use of health facilities for childbirth in Africa. Am J Public Health.

[B19] Lindelow M (2004). Understanding spatial variation in the utilization of health services: does quality matter?. Centre for the Study of African Economies: Working Paper Series.

[B20] Lindelow M (2005). The utilization of curative health care in Mozambique: Does income matter. J African Economies.

[B21] McLafferty SL (2003). GIS and health care. Annu Rev Public Health.

[B22] Diez-Roux A (2000). Multilevel analysis in public health research. Annu Rev Public Health.

[B23] MacNab YC (2003). Hierarchical Bayesian modeling of spatially correlated health service outcome and utilization rates. Biometrics.

[B24] Rushton G (2003). Public health, GIS and spatial analytic tools. Annu Rev Public Health.

[B25] Carter R, Mendis KN, Roberts D (2000). Spatial targeting of interventions against malaria. Bull World Health Organ.

[B26] Fahrmeir L, Lang S (2001). Bayesian Semiparametric Regression Analysis of Multicategorical Time-Space Data. Ann Inst Stat Math.

[B27] National Statistical Office, ORC Macro (2002). Malawi Demographic and Health Survey 2000.

[B28] Ben-Akiva M, Lerman S (1985). Discrete chocie analysis: Theory and Applications to Demand.

[B29] R Development Core Team (2004). R: A language and environment for statistical computing.

[B30] Besag J, York J, Mollie A (1991). Bayesian image restoration with two applications in spatial statistics (with discussion). Ann Inst Stat Math.

[B31] Brezger A, Kneib T, Lang S (2005). *BayesX*: Analyzing Bayesian structured additive regression models. J Stat Soft.

[B32] Slutsker L, Chitsulo L, Macheso A, Steketee RW (1994). Treatment of malaria fever episodes among children in Malawi: results of a KAP survey. Trop Med Parasitol.

[B33] Filmer D (2005). Fever and its treatments among the more poor and less poor In Sub-Saharan Africa. Health policy plan.

[B34] Kamat VR, Nichter M (1998). Pharmacies, self-medication and phamaceutical marketing in Bombay, India. Soc Sci Med.

[B35] Hausmann-Muela S, Ribera JM, Nyamongo I (2003). Health seeking behaviour and the health system response DCPP Working Paper no: 14.

[B36] Jensen ER, Stewart JF (2003). Health facility characteristics and the decision to seek care. J Devel Stud.

[B37] Foster S (1995). Treatment of malaria outside the formal health care. J Trop Med Hyg.

[B38] Hamel MJ, Odhacha A, Roberts JM, Deming MS (2001). Malaria control in Bungoma district, Kenya: a survey of home treatment of children with fever, bednet use and attendance at antenatal clinics. Bull World Health Organ.

[B39] Ettling M, Steketee RW, Macheso A, Schultz LJ, Nyasulu Y, Chitsulo L (1994). Malaria knowledge, attitudes and practices in Malawi: survey population characteristics. Trop Med Parasitol.

[B40] Fungladda W, Sornmani S (1986). Health behaviour, treatment seeking patterns, and cost of treatment for patients visiting malaria clinics in western Thailand. Southeast Asian J Trop Med Public Health.

[B41] Mugisha F, Kouyate B, Gbangou A, Saueborn R (2002). Examining out of pocket expenditure on health care in Nouna, Boukina Faso. Trop Med Int Health.

[B42] Deressa W, Ali A, Enqusellasie F (2003). Self-treatment of malaria in rural communities, Butajira, southern Ethiopia. Bull World Health Organ.

[B43] Dunyo SK, Koram KA, Nkrumah FK (1997). Fever in Africa and WHO recommendation. Lancet.

[B44] De Savigny D, Mayombana C, Mwageni E, Masanja H, Minhaj A, Mkilindi Y, Mbuya C, Kasale H, Reid G (2004). Care-seeking patterns for fatal malaria in Tanzania. Malaria J.

